# Fetal Alcohol Syndrome Prevention Research

**Published:** 2002

**Authors:** Janet R. Hankin

**Affiliations:** Janet R. Hankin, Ph.D., is a professor in the Department of Sociology, Wayne State University, Detroit, Michigan

**Keywords:** Fetal alcohol syndrome, prevention research, indicated prevention, selective prevention, universal prevention, targeted prevention, mass media prevention approach, public service announcement, warning label, prevention effort directed at people at risk, prevention outcome

## Abstract

Alcohol consumption during pregnancy can have numerous adverse health consequences for the developing fetus, including fetal alcohol syndrome (FAS) and alcohol-related effects, and therefore is a significant public health problem. A variety of programs have been developed to prevent drinking during pregnancy and the resulting health problems. Some of these efforts, such as public service announcements and beverage warning labels, are universal and strive to increase the public’s knowledge about FAS. Selective prevention approaches target women of reproductive age who drink alcohol. Such approaches may involve screening all pregnant women for alcohol consumption and counseling those women who do drink. Indicated prevention approaches target high-risk women (e.g., women who have previously abused alcohol or have had a child with FAS or other alcohol-related effects) and typically offer repeated counseling over several years. Both selective and indicated prevention efforts can reduce maternal alcohol consumption and improve the outcome of the offspring.

Drinking during pregnancy, which can result in serious birth defects, remains a significant public health problem despite a variety of prevention efforts that have been implemented in recent years. According to national data collected in 1999 by the Behavioral Risk Factor Surveillance System (BRFSS), a telephone survey of the noninstitutionalized U.S. population, 12.8 percent of pregnant women consumed at least one alcoholic drink during the past month, a decrease from 16.3 percent reported in 1995 (*a*Centers for Disease Control and Prevention [CDC] 2002*a*). The survey also assessed the prevalence of binge and frequent drinking (i.e., five or more drinks on one occasion or at least seven drinks per week) by pregnant women. Comparing data from 1995 and 1999, the investigators found that binge drinking and frequent drinking remained “substantially unchanged.” A total of 3.3 percent of pregnant women interviewed in 1999 reported frequent drinking and 2.7 percent reported binge drinking (CDC 2002*a*). These findings are subject to at least three limitations, however. First, BRFSS data are self-reported and might be subject to reporting biases, especially among pregnant women who are aware that alcohol use is not advised. Second, homeless women, women in homes without telephones, and women who were institutionalized were not surveyed. Both of these limitations could have an impact on prevalence rates. Third, because the proportion of pregnant women who were drinkers was limited in this sample, these estimated prevalence rates are subject to statistical limitations. Thus, the prevalence rates of drinking, frequent drinking, and binge drinking among pregnant women may actually be even higher than indicated in the BRFSS study.

The potential consequences of drinking during pregnancy—the most serious of which are fetal alcohol syndrome (FAS) and other manifestations collectively called alcohol-related effects—are preventable birth defects. Nevertheless, only limited evaluation research exists on FAS prevention programs (National Institute on Alcohol Abuse and Alcoholism [NIAAA] 2000*a*). After briefly describing the harmful effects of alcohol on the fetus, this article reviews the spectrum of FAS prevention efforts and summarizes recent research on FAS prevention activities.

## Consequences of Drinking During Pregnancy

Alcohol ingested during pregnancy can have a range of deleterious consequences for the developing fetus. The most severe condition caused by prenatal alcohol exposure is FAS, which is characterized by a particular pattern of facial anomalies, growth retardation, and developmental abnormalities in the central nervous system that often include, but are not limited to, mental retardation. Alcohol-related effects can be further subdivided into alcohol-related birth defects (ARBD) and alcohol-related neuro-developmental disorder (ARND). ARBD can involve defects in several organ systems, such as the heart, kidney, vision, and hearing. ARND manifests as central nervous system developmental abnormalities and/or behavioral or cognitive abnormalities. In addition, some evidence indicates that prenatal exposure to alcohol increases the risk for internalizing disorders, including depression and negative self-cognitions (e.g., low self-esteem) in the offspring (Olson et al. 2001). Furthermore, prenatal alcohol exposure may result in long-term neurocognitive disorders, such as problems with executive functions (e.g., poor organizational skills, difficulties in impulse control, and poor decisionmaking skills). Finally, adults who had been prenatally exposed to alcohol frequently suffer from mental disorders and maladaptive behaviors that make it difficult for them to be self-sufficient and independent (Streissguth and O’Malley 2000). Unfortunately, it is not uncommon for prenatal alcohol exposure to result in such severe deficits.

Estimates of the prevalence of FAS in the U.S. population range from 0.5 to 2 cases per 1,000 live births (May and Gossage 2001; CDC 2002*b*). Rates of FAS surpass this prevalence in high-risk populations. For example, reported rates of FAS are 9.8/1000 live births among Southwestern Plains Indians living on reservations (May and Gossage 2001). The rate for alcohol-related effects (ARBD and ARND) may be as high as 5 cases per 1,000 live births (Stratton et al. 1996). May and Gossage (2001) estimate that the prevalence for FAS, ARBD, and ARND combined is 1 percent of all births. The range in prevalence results from differences in the populations at risk being studied and in the methods used to identify affected people. Analyses based on medical records often underestimate the rates of FAS and alcohol-related effects compared with more aggressive case-finding approaches that include examinations of people living in the community (May and Gossage 2001; Stratton et al. 1996).

## The Spectrum of FAS Prevention Approaches

FAS can be prevented if a woman abstains from alcohol consumption at conception and throughout pregnancy. The Committee to Study Fetal Alcohol Syndrome of the Institute of Medicine of the National Academy of Sciences has described an intervention spectrum for FAS that includes three major prevention strategies (Stratton et al. 1996):

- *Universal prevention of maternal alcohol abuse.* These interventions attempt to educate the broad public about the risks of drinking during pregnancy. These universal efforts might be geared toward pregnant women or women of childbearing age and often include public service announcements, billboards, pamphlets in physicians’ offices, and media advertisements. The alcohol beverage warning label is an example of a universal intervention that has been extensively studied.
- *Selective prevention of maternal alcohol abuse.* These interventions target women who are at greater risk for having children with FAS or alcohol-related effects—that is, all women of childbearing age who consume alcohol. One example of selective prevention measures is the screening of all pregnant women for their alcohol use, followed by counseling of all drinkers regarding fetal risk or, if warranted, referral to specialized treatment.
- *Indicated prevention of FAS.* These measures are directed at high-risk women, including women who have previously abused alcohol while pregnant or while at risk for conception, or women who drink and have delivered an infant with FAS, ARND, or ARBD. This level of prevention includes alcoholism treatment of pregnant women or women who are likely to become pregnant as well as measures to encourage prevention of pregnancy.

The following sections describe each of these major types of FAS prevention efforts and summarize research on their effectiveness.

## Universal Efforts and Their Impact

One of the first steps in universal prevention efforts is to increase the public’s knowledge of the consequences of alcohol use during pregnancy, particularly FAS. Various methods can be used to increase knowledge, including news reports, articles in the popular press, public service announcements, billboards, and the alcohol beverage warning label. With the exception of the research on the warning label, few studies have assessed the effectiveness of these efforts on knowledge of FAS, attitudes about drinking during pregnancy, and women’s actual alcohol consumption during pregnancy.

### Extent of Media Attention to Drinking During Pregnancy

Lemmens and colleagues (1999) reviewed the coverage of alcohol-related issues in five national newspapers (i.e., *New York Times*, *Los Angeles Times*, *Washington Post*, *Christian Science Monitor*, and *Wall Street Journal*) from 1985 through 1991 by randomly sampling articles dealing with beverage alcohol. Out of 1,677 articles examined, only 23 dealt with alcohol and pregnancy. Similarly, Golden (2000) reviewed national network evening news broadcasts between 1977 and 1996 for ABC, CBS, NBC and found that alcohol and pregnancy was a topic in only 36 of the newscasts. These particular newscasts often coincided with the announcement of government warnings, the discovery of scientific evidence linking alcohol to birth defects, and other incidents associated with alcohol abuse deemed newsworthy, such as the firing of a bartender and waitress who refused to serve alcohol to a pregnant woman.

### Warning Posters

Warning posters to be placed where alcohol is sold have been required in some States as early as 1983. As of 1993, 18 States, 14 cities, and 2 counties required the display of such posters.

Prugh (1986) examined the impact of posters warning about drinking during pregnancy in New York City. Prior to the posters, 54 percent of respondents mentioned birth defects as a result of drinking while pregnant. A year after the posters were introduced, 68 percent mentioned birth defects as a consequence of drinking.

Using a national sample of 4,000 adults in 1990–1991, Kaskutas and Graves (1994) found that 31 percent of respondents saw a sign or poster warning about health effects of alcohol. Among those seeing a sign or poster, 56 percent recalled a warning about alcohol and birth defects. The investigators also reported that the level of knowledge of the risks associated with drinking during pregnancy increased with an increasing number of different message sources (e.g., posters, warning label, and advertisements). Among the 142 women in the survey who had been pregnant in the past year, 86 percent saw 1 or more messages about drinking while pregnant. Eighty-seven percent of women who had been pregnant versus 58 percent of women of childbearing age who had not been pregnant had a discussion about alcohol and the risk of birth defects (*p*<.05). Thirty-six percent of the women who had been pregnant and were drinkers reported limiting their drinking for “health reasons” compared with 25 percent of the non-pregnant women (*p*<.05). Finally, 70 percent of the women who had been pregnant reported that they did not drink alcohol while pregnant (Kaskutas and Graves 1994).

### Evidence that Knowledge of FAS Has Increased over Time

One study has tracked the level of knowledge of FAS over time using data from the National Health Interview Surveys that involved interviews with 19,000 people ages 18 to 44 in 1985 and with 23,000 people in 1990 (Dufour et al. 1994). Over the 5-year period between the two surveys, the proportion of respondents reporting that they had heard about FAS increased significantly, from 62 percent to 73 percent among women and from 49 percent to 55 percent among men. Among women who had heard of FAS, the number of those who correctly defined the condition as a birth defect increased significantly from 25 percent to 39 percent.

Among men, the percentages also increased significantly from 24 percent in 1985 to 36 percent in 1990.

Although this study did not test the effectiveness of particular universal interventions, the findings suggest that general knowledge of FAS has increased over time.

### Effectiveness of the Alcohol Beverage Warning Label

In 1988, the U.S. Congress passed the Alcoholic Beverage Warning Label Act requiring that effective November 18, 1989, a warning label must be attached to all containers of alcoholic beverages. The first part of the warning reads: “Government Warning: According to the Surgeon General, women should not drink alcoholic beverages during pregnancy because of the risk of birth defects.” Various researchers have examined exposure to the warning label and its impact on drinking during pregnancy. In general, the studies concluded that although awareness of the alcohol beverage warning label increased after the implementation of the law, this awareness has attenuated over time. Furthermore, the warning label’s impact on drinking during pregnancy has been modest. (For a comprehensive review of the impact of the alcohol warning label on perception of risks including drunk driving, birth defects, and health problems; and drinking behavior in a variety of situations, see Mackinnon 1995.)

For example, Greenfield and Kaskutas (1998) examined exposure to the warning label among a national probability sample of adults using annual cross-sectional telephone surveys.^1^ For that study, interviews were conducted in 1989, 1990, 1991, 1993, and 1994 that included a total of approximately 8,000 respondents. In 1990, 6 months after the implementation of the label, 21 percent of respondents said they had seen the warning label during the past 12 months. By 1994 exposure to the label had reached a plateau, according to the investigators, with 51 percent of respondents reporting that they had seen the label in the past 12 months.

As part of a cross-sectional and longitudinal study of the effects of alcohol beverage warning labels, Kaskutas and colleagues (1998) conducted a phone survey of a national representative sample of 365 pregnant women from 1989 through 1994. Exposure to the warning label fluctuated over the course of the study (7 percent saw the label in 1989, 27 percent in both 1990 and 1991, 58 percent in 1993, and 42 percent in 1994 [no data was collected in 1992]). Exposure to signs or posters also varied over the study period from a high of 28 percent in 1991 to a low of 13 percent in 1993 (1989, 21 percent; 1990, 17 percent; 1994, 17 percent). Advertisements about drinking during pregnancy were seen by 81 percent of women during 1989, 1990, and 1991, but by fewer women in 1993 and 1994 (65 percent and 58 percent, respectively). Finally, 84 percent of women had conversations about drinking during pregnancy in both 1989 and 1991, and 87 percent in 1990, but only 74 percent in 1993 and 58 percent in 1994. These data suggest changes, and in some cases, decreases in the proportion of women exposed to these media messages over time.

Seventy-five percent of the women reported not drinking, whereas 21 percent had one or two drinks and 4 percent admitted drinking at least three drinks on any single day during pregnancy. However, the 1989–1994 data showed no statistically significant relation between drinking patterns during pregnancy and exposure to any of the types of messages assessed in the surveys (Kaskutas et al. 1998).

Several other studies have tracked the awareness of warning labels in various populations, as follows:

- A Detroit study using a 1995 probability sample of 1,107 women found that 39 percent of the women had seen a warning label in the past 12 months. Among abstainers, 18 percent had seen a warning label, compared with 52 percent of women who drank. Seventy-seven percent of those who had seen the label recalled that it mentioned birth defects (Hankin 1998).
- An Indiana study evaluated knowledge of the warning label among 1,211 12^th^ grade students in the fall of 1989 (i.e., before the introduction of the label) and 2,006 students in the fall of 1990 (i.e., after the introduction of the label). The study found that in the fall of 1989, 26 percent reported having seen alcohol warning labels compared with 41 percent in the fall of 1990. In 1989, 65 percent of respondents who reported seeing the label also reported that it mentioned birth defects,^2^ whereas by the fall of 1990, this proportion had increased to 83 percent (Mackinnon et al. 1993).
- Another study tracked changes in label awareness from May 1989 through June 1993 among 7,334 inner-city African American women seeking prenatal care. Over the 50-month study period, the level of label awareness continued to increase through December 1992, when it reached the maximum of about 80 percent (Hankin et al. 1996).

Using the same inner-city prenatal clinic, Hankin and colleagues (1998) examined the impact of the warning label on drinking during pregnancy. This study involved 21,127 pregnant African American women using the prenatal clinic between 1986 and 1995. Controlling for patient characteristics and the unemployment rate,^3^ drinking began to decline 8 months after the implementation of the warning label (Hankin et al. 1998). However, this decline was only modest (i.e., 0.05 ounces of absolute alcohol per week or approximately 1 ounce of beer) and appeared to be short-lived. Thus, by 1992, the women’s alcohol consumption rose again and by 1995, pregnant women had become accustomed to the message.

## Selective Efforts and Their Impact

Selective prevention targets all women in their reproductive years who drink alcohol (although most studies target heavy drinkers).

One randomized trial assessed the impact of a brief intervention on drinking during pregnancy in this population (Chang et al. 1999, 2000). Women initiating prenatal care at Brigham and Women’s Hospital in Boston, MA, were screened for their alcohol use using a brief questionnaire called the T-ACE^4^ (Sokol et al. 1989). The first 250 women who were identified as risk drinkers using this questionnaire and who had consumed alcohol in the previous 6 months were randomly assigned to an assessment-only group (*n* = 127) or to a brief intervention group (*n* = 123). The brief intervention consisted of a 45-minute session with a physician and included the articulation of drinking goals while pregnant, identification of risk situations for drinking and alternatives to drinking, and the recommendation of abstinence during pregnancy from the Surgeon General and the Secretary of Health and Human Services. The study investigators then interviewed women once they had given birth about their alcohol consumption since the original assessment. Women in both groups reduced their alcohol consumption during pregnancy, and no difference existed between the two groups in the decrease in average number of drinks per drinking day. Accordingly, Chang and colleagues (1999) concluded that screening alone may be related to a reduction of drinking during pregnancy.

The study also attempted to identify patient characteristics that predicted greater success of the intervention approach. For example, the brief intervention appeared most successful for women who had been drinking alcohol in the previous 6 months but who had been abstinent in the 90 days prior to their first prenatal visit. Among current drinkers at baseline in the brief intervention group, women who articulated specific drinking goals for specific reasons were more likely to reduce alcohol consumption or abstain from alcohol during pregnancy than were women without such goals (Chang et al. 2000).

An ongoing randomized clinical trial is extending these selective prevention efforts by applying them to an indicated prevention program. In this trial, recruitment focuses on a high-risk population of 300 pregnant women who are currently drinking, drank during a previous pregnancy, or drank at least one drink daily prior to current pregnancy. In this study, the investigators, led by Chang, are comparing the results of an assessment-only condition with an enhanced brief intervention that involves a support partner chosen by the pregnant woman.

Handmaker and colleagues (1999) piloted a study to evaluate the results of motivational interviewing with 42 pregnant problem drinkers. Women reporting any recent drinking were randomly assigned either to the experimental group that received a 1-hour motivational interview focused on weighing drinking against the risk of birth defects, or to a control group that received a letter explaining the risks of drinking during pregnancy and recommending the woman talk to her obstetrical provider about the risks. Women in both groups had significantly reduced their alcohol intake at followup 2 months later. Women who self-reported the highest levels of blood alcohol concentrations had the greatest decrease in alcohol consumption if they were in the experimental group compared with the control group. (Blood alcohol concentrations were estimated using computer projections that were based on self-reports of estimated number of drinks, alcohol content of drinks, length of drinking episodes, the woman’s weight, and an average rate of alcohol metabolism for women.)

Another selective prevention approach that was part of the Developing Effective Educational Resources (DEER) project examined the exposure and reactions to warnings about drinking during pregnancy in samples of 321 pregnant Native Americans and African Americans living in the Northern California Bay area and Los Angeles. In this study, Kaskutas (2000) found that although the women were frequently exposed to warning messages, they were uncertain about the impact of FAS. Specifically, only about a quarter of the women could name at least one birth defect associated with FAS and only one-fifth knew that FAS was related to alcohol consumption.

Furthermore, the women did not understand the benefits of quitting drinking at any time during pregnancy, and they had the misconception that wine, beer, and wine coolers are safer to drink during pregnancy than liquor. Finally, most of the women underestimated their drinking. Thus, when the investigator compared alcohol intake using standard drink sizes^5^ with self-defined drink sizes (assessed with the help of beverage containers and photos), consumption by risk drinkers was 2 to 3 times higher using self-defined drink sizes compared with standard size drink measurements.

Ongoing research is extending this methodology and testing a novel prevention program for pregnant women enrolled in a health maintenance organization (Kaskutas and Graves 2001). In this randomized clinical trial, the investigators use models of alcoholic beverage containers (beverage containers of various sizes, such as 12-ounce versus 40-ounce beer bottles or beer cans, or liquor bottles that range from 375 milliliters, 750 milliliters, and 1 liter) or drinking vessels (shot glasses, wine glasses, or drinking glasses with lines marked off with letters so women could tell the investigators how high they filled the glass) and a computer program to help pregnant women understand how much they actually drink.

After the women identify the bottle or glass they typically drink from, the computer program calculates the absolute ounces of alcohol consumed. These nonconfrontational approaches of using drinking vessels and beverage containers and talking about drinking in a non-threatening way help the women discuss their drinking habits while pregnant.

## Indicated Efforts and Their Impact

Indicated prevention efforts are directed toward the population at highest risk of having children with FAS or alcohol-related effects—that is, women who have a history of drinking during pregnancy or have previously delivered a child affected by alcohol. Several studies have assessed prevention approaches directed at this population to prevent the birth of further alcohol-affected children. (Handmaker and Wilbourne [2001] thoroughly review motivational interventions in prenatal clinics, describing additional approaches not mentioned here.)

One of these approaches was the Protecting the Next Pregnancy project, which targeted women who had been identified as drinking heavily during the last pregnancy (called the index pregnancy). The goal of the intervention being tested was to reduce the women’s drinking during their next pregnancies (Hankin and Sokol 1995; Hankin et al. 2000). All women consuming at least four drinks per week (i.e., 0.3 ounces absolute alcohol per day) at the time they conceived during the index pregnancy were approached in the hospital’s postpartum unit and asked to participate in the trial. (The women’s average alcohol consumption was 1.2 ounces of absolute alcohol per day, or more than 16 drinks per week, at the time of conception for the index pregnancy.) Four weeks after giving birth, the women were randomly assigned to an experimental group that received an intensive brief intervention or a control group that received standard clinical care. The study included 300 women, who were followed up to 5 years.

The brief intervention involved a one-on-one method, which was based on a cognitive behavioral approach, and included 5 sessions beginning at 1 month after giving birth and continuing for 12 months. In those sessions, the counselor reviewed the definition of a standard drink, helped the women set the goal of abstention or reduction of alcohol use, established limits on consumption (if not abstaining), and taught ways to reduce drinking. Additional booster sessions were conducted over the 5-year followup period. The control group was simply advised that “You can have a healthier baby if you cut back or stop drinking during pregnancy.”

Of the 300 participants, 96 women delivered 1 or more infants during the followup period. The investigators found that women in the experimental group drank significantly less than did women in the control group during the subsequent pregnancies. While 25 percent of the women in the control group drank at least 0.3 ounces of absolute alcohol per day, only 11.8 percent of the women in the experimental group drank at that risk level (chi-square 2.4, *p* < .06, 1-tailed [Hankin and Sokol 1995; Hankin et al. 2000]). Furthermore, among women who drank during subsequent pregnancies, those from the experimental group drank about half as much as did women from the control group (i.e., 0.32 ounce versus 0.65 ounce absolute alcohol per day, *t* = 2.08, *p* < .03, 1-tailed). This reduced alcohol consumption resulted in improved birth outcomes among women from the experimental group, including fewer low-birth weight babies and fewer premature births. In addition, children born to women from the experimental group exhibited better neurobehavioral performance at 13 months of age compared with the children of women from the control group. These findings indicate that the brief intervention protected the next pregnancy by reducing alcohol consumption and improving infant outcomes.

In another indicated prevention effort called Project TrEAT (Trial for Early Alcohol Treatment) researchers screened almost 6,000 women ages 18 to 40 for problem drinking and then randomly assigned 205 problem drinkers to a brief intervention program or to a control group (Manwell et al. 2000). The two groups did not differ significantly with respect to various factors, such as alcohol use, age, socioeconomic status, smoking, various psychiatric disorders, lifetime drug use, or health care utilization. The brief intervention in this study consisted of two 15-minute counseling sessions conducted by physicians and including a review of the woman’s current health behavior, a discussion of the adverse effects of alcohol, a drinking agreement, and cards to record alcohol intake. The control group received a booklet on general health issues. Participants were followed for 48 months. Women in the brief intervention group successfully reduced their mean alcohol intake by 48 percent, and the proportion of women reporting any binge drinking in this group decreased from 93 percent to 68 percent. The control group also exhibited modest declines in alcohol use.

During the followup period, 41 women became pregnant, including 22 in the brief intervention group and 19 in the control group. For these women, the brief intervention seemed to result in better outcomes in terms of decreased consumption because women in the brief intervention group reduced their alcohol consumption from 13.6 to 3.5 drinks per week, compared with a decrease from 13.5 drinks to 10.1 drinks per week for women in the control group.

Another example of a treatment program targeting women who have already given birth to alcohol- or drug-exposed infants was the Seattle Birth to 3 Advocacy Project (Streissguth 1997). This program was designed for women who were heavy users of alcohol or other drugs, had no prenatal care, and were not connected to service providers during their pregnancy. Specially trained paraprofessionals,^6^ acting as advocates, worked on a one-on-one basis with the women and their families over a 3-year period. The 65 women in the program, most of whom were unemployed or on welfare, learned how to set goals, connect with other providers, and acquire new skills. After 2 years, 80 percent of the women had received alcohol and other drug abuse treatment, and 60 percent had remained abstinent from alcohol and other drugs. Moreover, 62 percent of the women were using long-term birth control methods, thereby reducing the risk for another alcohol-or drug-exposed pregnancy.

## Additional Examples of Ongoing FAS Prevention Research

Several other programs are studying different FAS prevention efforts in a variety of target populations and settings. For example, Project CHOICES (**C**hanging **H**igh-Risk Alc**O**hol Use and **I**ncreasing **C**ontraception **E**ffectiveness **S**tudy), which is funded by the Centers for Disease Control and Prevention (Floyd et al. 1999), is a selective prevention effort to prevent alcohol exposure during pregnancy among women of childbearing age in special settings. These populations include women in a jail, in a substance abuse center, or in clinics as well as a group of women with concerns about problem drinking who were recruited through media announcements. The program uses a brief intervention to reduce alcohol use and/or postpone pregnancy until drinking problems are resolved.

Another recently funded study is aimed at college students, encouraging them to abstain from alcohol or to use contraception if they drink. The goal of this program is to reduce alcohol use and promote effective contraception among women who are not currently pregnant. The program uses a brief intervention that educates women about the consequences of problem drinking, the benefits and costs of changing drinking and contraception behavior, setting goals, keeping a daily diary, and followup support.

An ongoing prevention effort on Native American reservations is based on the Institute of Medicine model and incorporates universal, selective, and indicated prevention activities (May 1995). The study includes four prevention communities and two “research only” communities. The selective prevention component consists of a screening program for women in clinics and Women, Infant, and Children (WIC) sites to identify high-risk drinkers. The indicated prevention component involves case management using motivational interviewing and community reinforcement approaches to help women who are drinking during pregnancy. The two sets of communities will be compared on a variety of outcome measures.

NIAAA is funding several other prevention studies of interventions designed to reduce drinking among pregnant women. (Information about these studies can be obtained from the Computer Retrieval Information on Scientific Projects [CRISP] database at http://crisp.cit.nih.gov.) Most of these efforts use brief interventions with motivational interviewing. Another program that is based at WIC clinics seeks to increase the detection of alcohol use during pregnancy, identify maternal characteristics contributing to the success of a brief intervention, identify characteristics of the intervention itself that contribute to its effectiveness, and evaluate the impact of the program on infant outcome. Finally, NIAAA is funding a study that is based on a more environmentally focused perspective and that examines the impact of alcohol server education in FAS prevention. All of these prevention efforts are ongoing, and researchers are still waiting for data on the results of these programs.

## Discussion

As noted by NIAAA, “Unfortunately, many women continue to drink during pregnancy. Furthermore, many of the women who continue to drink during pregnancy are at highest risk for having children with fetal alcohol syndrome and related problems. Thus, finding potent new ways to reach populations at risk and to influence changes in their behavior remains a challenge for alcohol research” (NIAAA 2000*b*, p. 3).

Researchers and clinicians already have made some progress in the efforts to prevent FAS. For example, universal prevention approaches have increased the general public’s knowledge about the results of drinking during pregnancy. Studies on awareness of the alcohol beverage warning label showed an increase in awareness over time. In addition, a larger proportion of the public knows about the relationships between drinking during pregnancy and birth defects. However, knowledge is not enough to change norms and actual behavior, as indicated by recent data that almost 13 percent of pregnant women drink during pregnancy (CDC 2002*a*). Numerous questions remain to be answered. For example, although the alcohol beverage warning label had a modest impact on drinking during pregnancy for a short time, the public has become habituated to its message. Future analyses need to clarify why this habituation occurred and whether new labels or a system of rotating labels can prevent habituation. Additional research must identify the most effective ways to educate the public about FAS (e.g., revised alcohol beverage warning labels, warning posters, public service announcements, or news reports). Systematic studies are needed that compare various universal prevention efforts and their impacts across various social groups.

Several researchers have examined the effects of selective and indicated prevention efforts using randomized clinical trials. The results described in this article suggest that brief interventions for pregnant women can successfully reduce alcohol intake during pregnancy. Additional studies using experimental designs (i.e., random assignment of study participants to an intervention group or to a control group that just receives standard clinical care) are necessary, however, to determine whether these findings are generalizable to pregnant women in diverse settings or whether the interventions need to be tailored to pregnant women from different ethnic and socioeconomic groups. Other unanswered questions concern the most appropriate contents for the brief intervention. Finally, it is important to understand whether the intervention results in clinically significant results across a variety of outcomes, including drinking during pregnancy, infant birth weight, length of gestation, and infant neurobehavioral outcomes. Although the research on the success of FAS prevention programs is still in its infancy, ongoing studies may help researchers and clinicians discover the best methods for separating alcohol from pregnancy and thus preventing FAS and alcohol-related effects.

## Footnotes

1 The term “probability sample” means that the sample was created to be representative of the U.S. population (e.g., included the same numbers of males, females, Blacks, Whites as the population). In a cross-sectional design, each participant is interviewed only once and a new sample is created for every year of the survey.

2 Reports of having seen warning labels before the labels actually existed are not uncommon. The explanation for such “false positives” is that subjects are likely to feel there must be a label if they are being asked about it.

3 Long-term drinking trends have been related to unemployment rates. For example, pregnant women may drink more when they have fewer resources and support. Furthermore, when unemployment is high, choices for prenatal care are limited, and more poor pregnant women may turn to the prenatal clinic where the study was conducted. Hankin and colleagues hypothesize that pregnant women may drink more when unemployment is high. They were unable to find any study that specifically examined this relationship. However, the following studies show that alcohol consumption, binge drinking, alcohol problems, and alcohol-related diseases are related to unemployment rates: Crawford, A.; Plant, M.A.; Kreitman, N.; and Latcham, R.W. Unemployment and drinking behaviour: Some data from a general population survey of alcohol use. *British Journal of Addiction* 82: 1007–1016, 1987; Brenner, M.H. Economic change, alcohol consumption, and heart disease mortality in nine industrialized countries. *Social Science and Medicine* 25:119–132, 1987; Linksy, A.S. ; Straus, M.S.; and Colby, J.P., Jr. Stressful events, stressful conditions, and alcohol problems in the United States: A partial test of Bale’s Theory. *Journal of Studies on Alcohol* 46:72–80, 1985; Catalano, R.; Dooley, D.; Wilson, G.; and Hough, R. Job loss and alcohol abuse: A test using data from the Epidemiologic Catchment Area Project. *Journal of Health and Social Behavior* 34:215–225, 1993.

4 The T-ACE consists of four questions and yields a maximum score of five points. Women who score two or more points are considered risk drinkers.

5 A standard drink frequently is defined as 12 ounces of beer, 5 ounces of wine, or 1.5 ounces of distilled spirits, each of which contains approximately 0.5 ounces (14 grams) of pure alcohol.

6 These were women with backgrounds similar to the clients’ (e.g., in terms of previous obstacles in their lives, such as alcohol use, poverty, single parenthood, or family violence) who had completed 2 years of college and had been trained in alcohol and other drug treatment, child development, parenting skills, and community resources.

## References

[b1-58-65] Centers for Disease Control and Prevention (CDC) (2002a). Alcohol use among women of childbearing age—United States, 1991–1999. Morbidity and Mortality Weekly Reports.

[b2-58-65] Centers for Disease Control and Prevention (CDC) (2002b). Fetal alcohol syndrome—Alaska, Arizona, Colorado, and New York, 1995–1997. Morbidity and Mortality Weekly Reports.

[b3-58-65] Chang G, Wilkins-Haug L, Berman S, Goetz M (1999). A brief intervention for alcohol use in pregnancy: A randomized trial. Addiction.

[b4-58-65] Chang G, Goetz MA, Wilkins-Haug L, Berman S (2000). A brief intervention for prenatal alcohol use: An in-depth look. Journal of Substance Abuse Treatment.

[b5-58-65] Dufour MC, Williams GD, Campbell KE, Aitken SS (1994). Knowledge of FAS and the risks of heavy drinking during pregnancy, 1985 and 1990. Alcohol Health & Research World.

[b6-58-65] Floyd RL, Ebrahim S, Boyle CA, Gould DW (1999). Preventing alcohol-exposed pregnancies among women of childbearing age: The necessity of a preconceptional approach. Journal of Women’s Health & Gender-Based Medicine.

[b7-58-65] Golden J (2000). “A tempest in a cocktail glass:” Mothers, alcohol, and television, 1977–1996. Journal of Health Politics, Policy and Law.

[b8-58-65] Greenfield TK, Kaskutas LA (1998). Five years’ exposure to the alcohol warning label messages and their impacts: Evidence from diffusion analysis. Applied Behavioral Science Review.

[b9-58-65] Handmaker NS, Wilbourne P (2001). Motivational interventions in prenatal clinics. Alcohol Research & Health.

[b10-58-65] Handmaker NS, Miller WR, Manicke M (1999). Finding of a pilot study of motivational interviewing with pregnant drinkers. Journal of Studies on Alcohol.

[b11-58-65] Hankin J (1998). Label exposure and recall among Detroit Metropolitan women. Applied Behavioral Science Review.

[b12-58-65] Hankin J, Sokol RJ (1995). Identification and care of problems associated with alcohol ingestion in pregnancy. Seminars in Perinatology.

[b13-58-65] Hankin JR, Sloan JJ, Firestone IJ (1996). Has awareness of the alcohol warning label reached its upper limit?. Alcoholism: Clinical and Experimental Research.

[b14-58-65] Hankin JR, Sloan JJ, Sokol RJ (1998). The modest impact of the alcohol beverage warning label on drinking during pregnancy among a sample of African American women. Journal of Public Policy and Marketing.

[b15-58-65] Hankin JR, McCaul ME, Heussner J (2000). Pregnant, alcohol abusing women. Alcoholism: Clinical and Experimental Research.

[b16-58-65] Kaskutas LA (2000). Understanding drinking during pregnancy among urban American Indians and African Americans: Health messages, risk beliefs, and how we measure consumption. Alcoholism: Clinical and Experimental Research.

[b17-58-65] Kaskutas LA, Graves K (1994). Relationship between cumulative exposure to health messages and awareness and behavior-related drinking during pregnancy. American Journal of Health Promotion.

[b18-58-65] Kaskutas LA, Graves K (2001). Pre-pregnancy drinking: How drink size affects risk assessment. Addiction.

[b19-58-65] Kaskutas LA, Greenfield T, Lee ME, Cote J (1998). Reach and effects of health messages on drinking during pregnancy. Journal of Health Education.

[b20-58-65] Lemmens PH, Vaeth PA, Greenfield TK (1999). Coverage of beverage alcohol issues in the print media of the United States, 1985–1991. American Journal of Public Health.

[b21-58-65] Mackinnon DP, Watson RR (1995). Review of the effects of the alcohol warning label. Alcohol, Cocaine, and Accidents: Drug and Alcohol Abuse Reviews.

[b22-58-65] Mackinnon DP, Pentz MA, Stacy AW (1993). The alcohol warning and adolescents: The first year. American Journal of Public Health.

[b23-58-65] Manwell LG, Fleming MF, Mundt MP (2000). Treatment of problem alcohol use in women of childbearing age: Results of a brief intervention trial. Alcoholism: Clinical and Experimental Research.

[b24-58-65] May PA (1995). A multiple-level comprehensive approach to the prevention of fetal alcohol syndrome (FAS) and other alcohol-related birth defects (ARBD). International Journal of Addiction.

[b25-58-65] May PA, Gossage JP (2001). Estimating the prevalence of Fetal Alcohol Syndrome: A summary. Alcohol Research & Health.

[b26-58-65] National Institute on Alcohol Abuse and Alcoholism (2000a). Tenth Special Report to the U.S. Congress on Alcohol and Health.

[b27-58-65] National Institute on Alcohol Abuse and Alcoholism (2000b). *Alcohol Alert* No. 50: “Fetal Alcohol Exposure and the Brain.”.

[b28-58-65] Olson HC, O’Connor MJ, Fitzgerald HE (2001). Lessons learned from study of the developmental impact of parental alcohol use. Infant Mental Health Journal.

[b29-58-65] Prugh T (1986). Point-of-purchase health warning notices. Alcohol Health & Research World.

[b30-58-65] Sokol RJ, Martier SS, Ager JW (1989). The T-ACE questions: Practical prenatal detection of risk drinking. American Journal of Obstetrics and Gynecology.

[b31-58-65] Stratton K, Howe C, Battaglia F (1996). Fetal Alcohol Syndrome: Diagnosis, Prevention, and Treatment.

[b32-58-65] Streissguth A (1997). Fetal Alcohol Syndrome: A Guide for Families and Communities.

[b33-58-65] Streissguth AP, O’Malley K (2000). Neuro-psychiatric implications and long-term consequences of fetal alcohol spectrum disorders. Seminars in Neuropsychiatry.

